# Foodborne Norovirus State of Affairs in the EU Rapid Alert System for Food and Feed

**DOI:** 10.3390/vetsci4040061

**Published:** 2017-11-25

**Authors:** Elias P. Papapanagiotou

**Affiliations:** Laboratory of Animal Food Products Hygiene-Veterinary Public Health, Department of Hygiene and Technology of Food of Animal Origin, School of Veterinary Medicine, Faculty of Health Sciences, Aristotle University of Thessaloniki, 54124 Thessaloniki, Greece; ipapapan@vet.auth.gr; Tel.: +30-231-099-9827

**Keywords:** norovirus, RASFF, FVO, INFOSAN, bivalve molluscs, fruits and vegetables

## Abstract

The European Union Rapid Alert System for Food and Feed (EU RASFF) database is an invaluable instrument for analyzing notifications involving norovirus in food. The aim of this work was to carry out a thorough research of the alert and border rejection notifications submitted in the RASFF database from its onset until 31 August 2017. Some conclusions of interest were: (i) Denmark, France, Italy, the Netherlands and Norway have contributed the majority of alert notifications as notifying countries, (ii) France and Serbia have been cited more often in alert notifications as countries of origin, (iii) Italy and Spain have submitted the majority of border rejection notifications, (iv) Third Countries implicated more frequently in border rejection notifications for norovirus in bivalve molluscs were Vietnam and Tunisia, whereas in fruits and vegetables were China and Serbia, (v) “risk dispersion” from norovirus-contaminated food was narrow since, in just over half of all alert notifications and all of the border rejection notifications, only up to three countries were involved, and (vi) both raw (oysters and berries) and cooked (mussels) food products can present a health risk to consumers. The information retrieved from the RASFF database on norovirus-contaminated food could prove helpful in the planning of future norovirus risk analysis endeavors.

## 1. Introduction

The European Union (EU) Rapid Alert System for Food and Feed (RASFF) was inaugurated in 1979 and represents a robust communication tool, enabling the fast distribution of important information between its members on risks found in food or feed, originating from within the EU or from a Third Country. The members of the EU RASFF are the 28 EU Member States, the European Commission Services, the European Food Safety Authority (EFSA), the countries of the European Economic Area (EEA), namely, Norway, Lichtenstein, Iceland and finally Switzerland [1].

In more detail, whenever one of the above-mentioned members identifies a risk contained in food or feed, it immediately notifies the RASFF in order to facilitate the instigation of risk management decision processes in other members of the system. Notifications vary according to their urgency in response as regards consumer health risks. The types of notifications that will be dealt with in the present study have been defined [2] as follows: (a) alert notifications are of an urgent nature in terms of the response required, the reason being that the food or feed item is already in the market and hence action must be taken immediately and (b) border rejection notifications are submitted from Border Inspection Posts (BIPs) when food or feed has been found to contain a clear risk to human health. Other types of notifications (not dealt with in this work) are information, information for attention and information for follow-up, which are also defined by the same EU legislation [2].

Noroviruses (belonging to the Caliciviridae family) are recognized as one of the most common causes of gastro-enteritis at the community level [3]. However, because the symptoms are relatively mild, infections go largely underreported, except during major outbreaks, especially those in health care institutions such as nursing homes and hospitals, and, in high-risk patients, may demonstrate more severe symptoms [4]. They cause an acute, self-limiting gastroenteritis and are characterized by frequent vomiting, abdominal cramps and diarrhea. In recent years, foodborne noroviruses have been linked to some significant, sometimes large foodborne outbreaks e.g., an outbreak in Germany affecting approximately 11,000 people that was linked to frozen strawberries [5].

Foodborne transmission is likely to occur directly through food vehicles such as bivalve molluscs and soft fruit, through contaminated food handlers and preparation surfaces. Both food- and water-borne transmission can occur, but the relative importance of these routes compared to person-to-person spread is largely unknown. The general consensus is that the consumption of shellfish is associated world-wide with an increased risk of viral infection. Noroviruses are also well documented in fresh produce, with large outbreaks (often international) reported to be related to the consumption of contaminated berries [6].

Viruses in general need living cells to replicate, and almost all food-borne viruses are strictly human pathogens. This means that the transmission via food reflects fecal contamination with the persistence of viruses on or in the product, but no replication. Noroviruses can accumulate and concentrate in the guts of oysters growing in sewage-contaminated waters and indeed, noroviruses have been found to persist in oysters and are difficult to eliminate from contaminated oysters due to their specific attachment to oyster tissues such as the gills and digestive glands. This binding enhances the survival of norovirus in oysters due to the favorable environmental condition in oyster tissues [7]. Depuration and relaying are processes often used in bivalve molluscs to remove bacterial enteric pathogens from contaminated animals. In the case of noroviruses, the aforementioned approaches are not as efficient, hence the foodborne outbreaks especially from oysters that are usually consumed raw.

Most noroviruses that infect humans belong to genogroups GI and GII. Noroviruses of both GI and GII have been detected in oysters and in stool samples collected from cases in outbreaks. This is a common finding in oyster-related outbreaks and reflects an environmental source of contamination. Up to seven different strains of norovirus in some outbreaks have been reported [8]. This contrasts with person-to-person transmission occurring in settings such as hospitals or nursing homes, which most often involve a single genotype [9]. GII.4 strains have the highest prevalence in the winter season in temperate regions. Despite their sensitivity to humidity human noroviruses are very stable in water [10]. The control of food-borne viral diseases is challenged by the detection of viruses in foods. Such detection is largely dependent on molecular techniques. Quantitative Reverse Transcriptase Polymerase Chain Reaction (RT-qPCR) is the analytical method of choice. Although TaqMan-based RT-PCR assays continue to serve as robust methods for the sensitive detection of noroviruses, new technologies such as next generation sequencing [11] and digital PCR [12,13,14] have become available and have great potential for detection and typing of these viruses including in environmental samples [15].

The major barrier to research and development of effective interventions for human noroviruses has been the lack of a robust and reproducible in vitro cultivation system. Such a system is critical to achieve a full mechanistic understanding of human noroviruses replication, stability, evolution and pathogenesis. Recently, stem-cell derived, non-transformed human intestinal enteroid (HIEs) cultures validated as an appropriate pre-clinical model for clinically important enteric infections have been reported [16,17].

Historically, systematic surveillance for food-borne viral diseases has been implemented by the Food-borne Viruses in Europe (FBVE) network, which was a joint electronic database to facilitate data comparison and harmonize strain nomenclature. NoroNet is a continuation of the FBVE network, involving 13 European countries existing since 1999 who share surveillance and research data on enteric virus infections, focusing mainly on norovirus. The work of the network has been supported through various EU projects [18]. In the Netherlands, norovirus remains the key pathogen causing food-related outbreaks in 2016 as in previous years, followed by Salmonella and Campylobacter [19]. Interestingly, new variants of GII.4 emerged in 2002, 2004 and 2006; each time displacing the resident virus population within months across Europe, indicating viral evolution [20].

The European Food Safety Authority (EFSA) has produced very significant opinions regarding norovirus [21,22] and has evaluated berries vis-a-vis norovirus as well [23]. The EFSA was asked to deliver a scientific opinion on the evaluation of heat treatments, different from those currently established in the EU legislation that could be applied to live bivalve molluscs. Of particular relevance are the achievement of at least 90 °C for at least 90 s in the mollusc flesh and the inactivation of viruses [24]. EFSA has also reported on technical specifications of a baseline survey in oysters [25] and some “strong-evidence food-borne outbreaks” caused by viruses in the EU in 2015 [26].

In accordance to the general obligation to produce safe food, food business operators (FBO) are encouraged to work with the relevant competent authorities (CA) to develop guidance documents on managing norovirus risk in oysters, including practical strategies for reducing norovirus concentrations in oysters. People who are immuno-compromised or otherwise vulnerable to infection should be advised against consuming uncooked oysters. Routine monitoring of norovirus levels in oysters before they are placed on the market is not legally required at present [27].

At the request of the European Commission for scientific technical assistance, a European Union-coordinated monitoring programme on the prevalence of norovirus in raw oysters was initiated. The objective of the study was to estimate the European prevalence of norovirus-contaminated oysters at production areas and batches of oysters at dispatch centers, with a 95% level of confidence and a level of precision of 5% considering an expected prevalence of 50%. The survey started in November 2016 and finishes in October 2018 [28]. The role of the EU Reference Laboratory (EURL) for monitoring bacteriological and viral contamination of bivalve molluscs, namely, Centre for Environment, Fisheries and Aquaculture Sciences (CEFAS) [29] in support of the sampling plan whose timing would be bi-monthly to take into account temporal variability, is quite significant.

The Food and Veterinary Office (FVO) of the European Commission has a pivotal role to play in the safeguarding of human health [30]. It interacts with the RASFF in such a way that whenever an important risk appears in foods (of both animal and non-animal origin) that threatens the health of the consumers, action in the form of audits and inspections is initiated. The European Centre for Disease Prevention and Control (ECDC) has been actively involved in the norovirus issue by providing tools that can prove useful during an investigation of a food- and waterborne outbreak in Europe [31].

To accommodate the goals of disseminating food safety information to various stakeholders in the food chain continuum and improving national and international collaboration of the International Network of Food Safety Authorities (INFOSAN), partnerships with other networks, initiatives and agencies have been established [32]. These partnerships enable the efficient exchange of incident and emergency information and support the establishment of one global system for the exchange of information during international food contamination and foodborne disease events of international concern.

Furthermore, in a recent Food Standards Agency (FSA) Conference [33], some very interesting proposals were made for ongoing research efforts that were focusing on the following areas: (i) whole genome sequencing for the characterization of norovirus and other foodborne viruses; (ii) surveillance to generate more information about levels of norovirus occurring in food; (iii) refinements to current RT-PCR to improve detection of low numbers of norovirus particles in all food matrices; (iv) the binding properties and possible methods of inactivation of norovirus; (v) the effectiveness of depuration (or alternatives such as high pressure, UV, ozone, irradiation) in removing norovirus from oysters; and (vi) establishment of the infectious dose in different food commodities including shellfish and fresh produce (lettuce and berries).

A systematic work to explore the EU RASFF database for the purpose of supporting risk analysis of biogenic amines has already been published [34]. This excellent work was based on the evolution of RASFF notifications, in anticipation that these data would offer true proof to bolster food safety risk analysis for future purposes.

In the present study, an attempt to describe in detail the situation of norovirus-related notifications in the EU RASFF from its beginning until the 31st of August 2017 was realized. In this way, the whole picture of the state of affairs concerning the norovirus issue throughout the entire EU RASFF existence period could be highlighted. This information could prove useful for future interventions on the part of the FVO and other EU Institutions as well provide opportunities for enhanced collaboration between EU MS within the RASFF and with INFOSAN of the World Health Organisation (WHO). Lastly, all relevant stakeholders could greatly benefit from sharing this information emerging from the EU RASFF in the greater context of future risk analysis purposes.

## 2. Materials and Methods

The search in the EU Rapid Alert System for Food and Feed database (https://webgate.ec.europa.eu/rasff-window/portal/?event=searchForm&cleanSearch=1) [35] retrieved the data used for this research (accessed on 1 September 2017). The following criteria were used in the search undertaken for subject “norovirus” (from 1979 till 31 August 2017): food, notifying country, country of origin, food product category, year of notifications, and also month of notification, type of notification, classification basis, risk decision, distribution, etc. All data were extracted in Excel files in the various combinations chosen. In addition, data were retrieved on alert notifications for other hazards (“Salmonella” and “Listeria”) within the “pathogenic microorganisms” food product category, for comparative purposes. In the context of this paper, only alert and border rejection notifications will be examined.

## 3. Results

### 3.1. Alert and Border Rejection Notifications in Food with “Norovirus” Submitted until 31 August 2017 in Bivalve Molluscs

Historically, the inaugural alert notification implicating “norovirus” in “food” in bivalve molluscs in the EU RASFF database was submitted by the Netherlands on 16 February 2001 under the hazard category “pathogenic microorganisms”, the product category “molluscs and products thereof (obsolete)” and concerned oysters. The very first alert notification that involved “norovirus” in fruits and vegetables was submitted by Denmark on 3 June 2005 and was norovirus (suspicion) in frozen raspberry crumble. Since then, numerous alert notifications have been submitted in the RASFF database concerning norovirus in food product categories of both animal and non-animal origin, all under the “pathogenic microorganisms” hazard category.

The first border rejection notification ever to be submitted to the EU RASFF database in bivalve molluscs was in frozen scallops (on 24 June 2009) and in fruits and vegetables was in frozen blackberries (on 1 April 2011).

The alert notifications implicating “norovirus” presented in Table 1 were made on the basis of: (i) “official control on the market” (official control on the EEA internal market); (ii) “company’s own check” (notification initiated through a company notifying the outcome of an own-check to the competent authority); (iii) “consumer complaint” (notification initiated through a consumer lodging a complaint with the competent authority); and (iv) “food poisoning” (reports of a food poisoning leading to the notification of a risk in a food on the market that has caused the food poisoning).

Table 1 shows the allocation of alert and border rejection notifications for “norovirus” in various food product categories throughout the examined period (from the onset of the RASFF until 31 August 2017). It should be mentioned here that four alert notifications were assigned to an obsolete today, food product category, namely, “molluscs and products thereof (obsolete)” and were all submitted for “norovirus” presence in oysters. In addition, another five notifications under the food product category “crustaceans and products thereof” were all submitted for “norovirus” presence in oysters. Since both of the aforementioned food product categories were notifications in oysters, for the purposes of this study, they were included in the 52 alert notifications cited from “bivalve molluscs and products thereof” food product category. Thus, from this point onwards, the food product category “bivalve molluscs” encompasses the results from the three categories.

All in all, the 61 alert notifications reported in the EU RASFF that involved “norovirus” in bivalve molluscs were notified by the following EU RASFF members, namely Italy (*n* = 13), Norway (*n* = 11), Denmark (*n* = 10), France (*n* = 9), the Netherlands (*n* = 9), Ireland (*n* = 5), Spain (*n* = 2), Malta (*n* = 1) and Germany (*n* = 1). Oyster (*n* = 51) was the bivalve mollusc most often cited in the alert notifications, but also clams (*n* = 4), mussels (*n* = 4) scallops (*n* = 1) and one notification that generally cited “bivalve molluscs”. France (*n* = 35) was the EU RASFF member country most often cited, as the country of origin in alert notifications.

The 37 border rejection notifications reported in the EU RASFF that involved “norovirus” in bivalve molluscs were notified by two EU RASFF MS by and large, namely, Italy (*n* = 17) and Spain (*n* = 12). Clam (*n* = 35) was the bivalve mollusc most often cited but scallop (*n* = 2) was also cited and the Third Countries most frequently notified as the countries of origin were Vietnam (*n* = 19) and Tunisia (*n* = 10).

### 3.2. Alert and Border Rejection Notifications in Food with “Norovirus” Submitted until 31 August 2017 in Fruits and Vegetables

The 46 alert notifications reported in the EU RASFF database that involved “norovirus” in fruits and vegetables (Table 1) were most frequently notified by two EU RASFF member countries, namely, Denmark (*n* = 17) and France (*n* = 10). In addition, Finland (*n* = 4), Sweden (*n* = 4), the Czech Republic (*n* = 3), the Netherlands (*n* = 3), Belgium (*n* = 2), Germany (*n* = 2), and the United Kingdom (*n* = 1) contributed such notifications. Raspberries (*n* = 31) was the fruit most often cited while strawberries (*n* = 5), blueberries (*n* = 2), blackberries (*n* = 1), lingonberries (*n* = 1), forest fruit mix (*n* = 1) as well as lettuce (*n* = 2), were also cited. Additionally, three alert notifications cited tomatoes (e.g., cherry tomatoes, cocktail tomatoes and dried tomatoes in sunflower oil) for “norovirus” presence and were all notified by the same member country, namely the Czech Republic in 2017. Serbia (*n* = 17) and Poland (*n* = 14) were the countries most often cited as the countries of origin in such notifications with others being much less frequently cited such as China (*n* = 3), Chile (*n* = 3), Ukraine (*n* = 2), Morocco (*n* = 2), etc.

The five border rejection notifications reported in the EU RASFF that involved “norovirus” in fruits and vegetables involved strawberries (*n* = 3), blackberries (*n* = 1) and raspberries (*n* = 1). The notifying countries were Denmark (*n* = 2) and Hungary, Lithuania and Finland (*n* = 1 each), whereas the Third Countries cited as the countries of origin were China (*n* = 3) and Serbia (*n* = 2).

### 3.3. Alert Notifications in Food with “Norovirus” Submitted until 31 August 2017 by the EU RASFF Member Countries as Notifying Countries and as Countries of Origin

**Figure 1:**
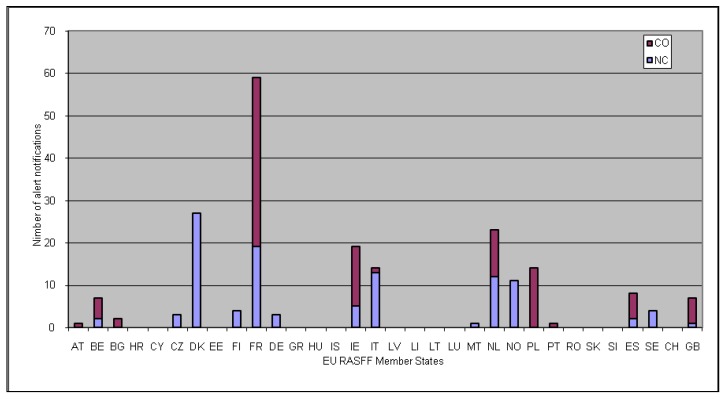
The European Union Rapid Alert System for Food and Feed (EU RASFF) members acting as notifying countries (NC) and countries of origin (CO) for “norovirus”-related alert notifications in food generally, until 31 August 2017 in the EU RASFF database (AT: Austria, BE: Belgium, BG: Bulgaria, HR: Croatia, CY: Cyprus, CZ: Czech Republic, DK: Denmark, EE: Estonia, FI: Finland, FR: France, DE: Germany, GR: Greece, HU: Hungary, IS: Iceland, IE: Ireland, IT: Italy, LV: Latvia, LI: Liechtenstein, LT: Lithuania, LU: Luxembourg, MT: Malta, NL: Netherlands, NO: Norway, PL: Poland, PT: Portugal, RO: Romania, SK: Slovakia, SI: Slovenia, ES: Spain, SE: Sweden, CH: Switzerland, GB: United Kingdom).

The EU RASFF members could be divided into four distinct categories according to their activity logged in the RASFF database, in their capacities both as notifying countries and countries of origin, regarding alert notifications, as follows: (i) RASFF members that submitted any number of alert notifications as notifying countries and, at the same time, any number of alert notifications involved them as countries of origin (e.g., Belgium, France, Ireland, Italy, the Netherlands, Spain, the United Kingdom); (ii) RASFF members that submitted any number of alert notifications as notifying countries but were not cited in alert notifications as countries of origin at all (e.g., Czech Republic, Denmark, Finland, Germany, Malta, Norway, Sweden); (iii) RASFF members that did not submit any alert notifications as notifying countries but were cited in alert notifications as countries of origin (e.g., Austria, Bulgaria, Poland, Portugal); and (iv) RASFF members that did not submit any alert notifications at all neither as notifying countries nor were they cited in alert notifications as countries of origin (e.g., Croatia, Cyprus, Esthonia, Greece, Hungary, Iceland, Latvia, Lichtenstein, Lithuania, Luxembourg, Romania, Slovakia, Slovenia, Switzerland). The status of notifying country and country of origin for all EU RASFF member countries is shown in Figure 1, concerning alert notifications implicating “norovirus” presence in food, until 31 August 2017.

### 3.4. Temporal (Monthly and Yearly) Distribution of Alert and Border Rejection Notifications in Food with “Norovirus” Submitted until 31 August 2017 in the EU RASFF

The actual month that the majority of alert notifications on “norovirus” in bivalve molluscs were reported, was March (*n* = 18) with February and January following in terms of frequency of notifications. Interestingly, it was March (*n* = 10) that border rejections in bivalve molluscs were reported most often as well. Regarding alert notifications on “norovirus” in fruits and vegetables, June was the month that the majority of alert notifications were reported (*n* = 8), whereas a clear peak in any single month was not evident in the case of border rejection notifications since only one such notification was reported in five different months.

The alert notifications concerning “norovirus” in bivalve molluscs exhibited a peak in 2013 (*n* = 11) and proceeded with a decreasing trend until 2017, whereas in border rejection notifications the peak occurred in 2004 (*n* = 27) with a clearly decreasing trend until 2017. In alert notifications concerning “norovirus” in fruits and vegetables, the peak was noted in 2017 (*n* = 10) and in border rejection notifications in 2013 (*n* = 2). From the RASFF database search, sampling dates were not available in 28 alert and three border rejection notifications, while notification dates were always provided. This is the reason why the latter were selected for the monthly distribution search in this work, although the use of sampling dates would have been more representative.

### 3.5. “Countries/Organizations Involved” in Alert and Border Rejection Notifications and “Distribution Status” in Food with “Norovirus” Submitted until 31 August 2017 in the EU RASFF

In the EU RASFF database, the number of countries/organizations involved in each notification varied significantly in alert and border rejection (Table 2). More specifically, alert notifications that involved ≥10 countries (namely 19, 16, 12, 11 and 10 countries) were cited once, once, once, three and two times, respectively.

The “distribution status” indicates knowledge existing at the time of notification about the possible distribution of the product on the market. The concept “market” is to be interpreted in a geographical way: “the European Economic Area (EEA) market”, i.e., this does not necessarily mean that the product is already on the shelves available to consumers, as often it is not.

The most frequently cited “distribution status” of food contaminated with “norovirus” in alert notifications in both bivalve molluscs and fruits and vegetables was reported as following: “distribution to other member countries” (*n* = 42), “distribution on the market (possible)” (*n* = 26), “distribution restricted to notifying country” (*n* = 12), and “no distribution from notifying country” (*n* = 10).

As regards border rejection notifications, the most frequently mentioned “distribution status” of “norovirus”-contaminated food (both bivalve molluscs and fruits and vegetables) was “product not (yet) placed on the market” (*n* = 37), “no distribution” (*n* = 4) and “product allowed to travel to destination under customs seals” (*n* = 1).

### 3.6. Risk Decision Categorization of Alert and Border Rejection Notifications in Food with “Norovirus” Submitted until 31 August 2017 in the EU RASFF

The EU RASFF database provides information on the risk decision made on a single notification that being either “serious” or “not serious” or “undecided”. The very first alert and border rejection notifications to be designated as “serious” (in terms of risk decision) were submitted to the RASFF database in 27 June 2012 and in 18 November 2011. Regarding food contaminated with “norovirus” the respective categorizations are shown in Table 3. 

It should be mentioned here that the categorization of the notifications made on the basis of risk decision does not apply to all notifications submitted in the EU RASFF database on “norovirus”, since it has been in force since 2012.

### 3.7. Relative (%) Frequency of Citation of Alert Notifications, Alert Notifications Made on the Basis of “Food Poisoning”, Alert Notifications with a “Serious” Risk Decision to All Alert Notifications in Food with “Norovirus” Submitted until 31 August 2017 in the EU RASFF

The relative (%) frequency of citation of (i) alert notifications on “norovirus” to all types of notifications on “norovirus”, (ii) alert notifications made on the basis of “food poisoning” on “norovirus” to all alert notifications on “norovirus” and (iii) alert notifications made on the basis of “food poisoning” on “norovirus” to all alert notifications made on the basis of “food poisoning” are shown in Table 4. Additionally, the percent frequency of citation of alert notifications on “norovirus” with a “serious” risk decision to all alert notifications on “norovirus” within the hazard category “pathogenic microorganisms” in food and finally for comparative purposes, the respective percent frequencies of citation of other important pathogenic microorganisms, e.g., “Salmonella” and “Listeria” in the “pathogenic microorganisms” hazard category have also been included in Table 4.

“Food poisoning” as a reason for notification, which was introduced in the RASFF database in 2008, can be anticipated to become increasingly valuable for risk assessment and management [34] by providing a direct link between occurrence data of hazards such as norovirus in notified food and adverse health effects of consumers. The very first alert notification of “food poisoning” implicating “norovirus” in food (oysters) was submitted to the RASFF database by the Netherlands on 24 January 2008), under the food product category “bivalve molluscs and products thereof”. 

### 3.8. Genogroups Identified in Alert and Border Rejection Notifications in Food with “Norovirus” Submitted until 31 August 2017 in the EU RASFF and State of Food Reported in the RASFF Database

Unfortunately, not all alert and border rejection notifications reported in the RASFF database specifically mentioned the actual genogroups of “norovirus” that were identified in either bivalve molluscs or fruits and vegetables. In those alert notifications in both food product categories in which genogroups were mentioned, the citation frequency (in descending order) was GII > GI and GII together > GI, whereas the remaining were usually reported as “presence” or “positive”, etc. The respective genogroups’ frequency of citation in the border rejection notifications was (in descending order) the following: GII > GI > GI and GII together, but the majority were usually reported as “presence” or “positive”, etc.

Finally, in alert and border rejection notifications concerning bivalve molluscs, raw, fresh chilled, frozen, frozen (pre)cooked oysters and live, chilled, frozen, frozen cooked/boiled/blanched clams have been described in the RASFF database. As far as fruits and vegetables are concerned, in alert and border rejection notifications, frozen raspberries, raspberry sauce, dried tomatoes and frozen raspberries have been notified to be containing noroviruses.

## 4. Discussion

In direct accordance with the relevant literature, food of animal origin such as bivalve molluscan shellfish present a particularly high risk because of their ability to concentrate viruses from contaminated waters [36] and have been reported in the EU RASFF to be contaminated with “norovirus”. Additionally, food of non-animal origin such as various types of berries and lettuce, have also been reported in the RASFF as being vehicles of transmission for “norovirus”. Interestingly, tomatoes contaminated with “norovirus” were reported in 2017 and this exemplifies the important role of the EU RASFF in revealing emerging issues that require scientific attention vis-à-vis food safety.

In the EU RASFF, the first alert notifications citing “norovirus” in bivalve molluscs and fruits and vegetables date back to 2001 and 2005, respectively, and the first border rejection notifications were submitted in the EU RASFF in 2009 and 2011, respectively. This shows that “norovirus” is a relatively “new acquaintance” in terms of citation history in RASFF border rejection notifications (involving Third Countries as countries of origin) but a rather “old acquaintance” in alert notifications. The first time that “Salmonella” was cited in alert and border rejection notifications was on 28 July 1982 and 30 January 2008, respectively, whereas the respective dates for “Listeria” were 31 May 1986 and 24 February 2009. This indicates that “norovirus” is apparently a relatively “new” hazard in the EU RASFF database, in alert notifications. Of course, this holds true strictly within the context of the examined RASFF database information available, since norovirus perhaps was not regularly recorded as routine diagnostic tools were not as widely available.

The relative frequency of citation of alert notifications on “norovirus’ over all types of notifications on “norovirus’ within the hazard category “pathogenic microorganisms” in relation to the respective frequencies of other major foodborne biological hazards, in descending order was: “Listeria” > “norovirus” > “Salmonella”. Furthermore, the relative frequency of citation of alert notifications on “norovirus’ made on the basis of food poisoning over all alert notifications on “norovirus’, places “norovirus” well ahead of “Salmonella” and “Listeria”. Additionally, the relative frequency of citation of alert notifications on “norovirus’ made on the basis of food poisoning over all alert notifications made on the basis of food poisoning ranks “norovirus” ahead of both “Salmonella” and “Listeria”. Finally, the relative frequency of citation of alert notifications on “norovirus’ designated as having a “serious” risk decision over all alert notifications on “norovirus”, ranks norovirus ahead of “Salmonella” and “Listeria”. The aforementioned ranking of “norovirus” is indicative of the significance of these foodborne viruses have surmounted over the years, in relation to other very important biological hazards within the EU RASFF.

The contribution to alert and border rejection notifications on the part of RASFF member countries as notifying countries, is evidently not balanced, but, in all fairness, one has to take into consideration the diversification in animal production and trade volumes as well as food market dynamics, specialized eating habits or traditional preferences of the people in each member country and the history with international trade partners, over the years. Additionally, some countries may be the major points of entry into the EEA of specific food products from various Third Countries (TC). For example, in border rejection notifications, the following Third Countries (acting as countries of origin) have been cited to have exported bivalve molluscs consignments to specific EU RASFF members. France has been the sole recipient of such food contaminated with “norovirus” from Peru, whereas Italy has received respective food consignments from Turkey and Tunisia. An exception to the above pattern is Vietnam that has exported to Spain, Portugal and Italy (showing a more diversified spectrum regarding countries of export).

In the same context, there appear to be notifying member countries that exhibit activity in the EU RASFF by submitting alert notifications for “norovirus” only in bivalve molluscs (Italy, Ireland, Spain, Malta, Norway) or only in fruits and vegetables (Finland, Sweden, the Czech Republic, Belgium, Great Britain) or in both product categories (Denmark, France, the Netherlands, Germany). The situation in border rejection notifications is somewhat different since the notifying member countries for “norovirus” in bivalve molluscs (Italy, Spain, Portugal, France) are different than those for “norovirus” in fruits and vegetables (Finland, Hungary, Denmark, Lithuania). In effect, through the above description, one can decipher the fundamental rationale behind the RASFF, where some countries protect the rest of the countries of the network from specific hazards contained in specific food.

The monthly dispersion of “norovirus”-related alert and border rejection notifications in bivalve molluscs present an overall peak in March when all notification dates of all notifications in the EU RASFF database are taken into consideration, whereas, for fruits and vegetables, the peak for alert notifications is June while no peak was noted for border rejection notifications. The yearly distribution of “norovirus”-contaminated bivalve molluscs and fruits and vegetables shows a peak in 2013 and 2014, and 2017 and 2013, respectively.

The “risk dispersion” from “norovirus”-contaminated food was rather narrow since not a great number of countries were involved, as a general rule, in the alert and border rejection notifications cited in the EU RASFF database. Up to three countries were involved in just over half the alert notifications and all the border rejection notifications. Consequently, this could prove to be quite helpful when and if a traceability emergency is deemed necessary under a food crisis situation and remediation/mitigation intervention measures are required to be implemented urgently.

Of equal importance is the fact that the food product categories involved in “norovirus”-related alert notifications are only two, which is reassuring when compared with other significant hazards within the hazard category pathogenic microorganisms such as “Salmonella” and “Listeria” whose respective numbers of food product categories are 23 and 16. The respective spectrum of “risk dispersion” in food product categories, for border rejection notifications is the following: “norovirus” (*n* = 2), “Salmonella” (*n* = 14) and “Listeria” (*n* = 3). Nevertheless, this could be a misleading low number, and there is the possibility that norovirus is being underreported and there could possibly be other food categories that could be relevant.

The status of food implicated in “norovirus”-related EU RASFF alert and border rejection notifications was fresh, chilled, frozen, cooked and still retained its ability to exert infectivity on the consumer. This is clearly indicative of the fact that either raw (e.g., oysters and berries) or cooked (e.g., mussels) or dried (e.g., tomatoes) the food product categories involved (when contaminated with “norovirus”) can present a clear and definite health risk to consumers. Most outbreaks of shellfish-associated norovirus disease are linked to oyster consumption because they are eaten usually raw, but some outbreaks have also been linked to cooked oysters [37].

With regard to the genogroups reported in the RASFF database, the revealed trend is for GII to prevail, but bearing in mind that the majority of notifications (both alert and border rejection) did not have an identification result down to the genogroup level and presented their results as merely “presence” or likewise. In literature, it has been reported that GI strains are more frequently encountered in shellfish-related outbreaks and the GII.4 genotype is not as dominant [38].

As early as 2004, it was proposed that, for the control of foodborne viral infections (amongst other interventions), emphasis should be put on food safety quality control and management systems (GHP, GMP, HACCP) [39]. It seems inevitable that some form of a means of disinfection (partial or complete to diminish or eliminate contamination) is a clear prerequisite that needs to be addressed. Quite possibly, more than one option would have to be available at hand for application to the different types of food in question, namely, shellfish and soft fruits. Until this goal is attained, good hygienic (pre-harvest food safety) management practices need to be followed closely in order to minimize viral contamination at the initial stages of production. The FAO/WHO Codex Alimentarius [40] has already offered a thorough insight into the control of norovirus in bivalve molluscs and fresh produce in the context of a science-based approach.

All the aforementioned information ought to be put into the perspective of a risk-based integrated approach, since no information on the amount of consignments contaminated with “norovirus” is given in the EU RASFF database, and also since the contamination of food with “norovirus” is mainly reported qualitatively as “presence”, one could not conclude on the actual risk level the consumers might have been confronted with.

A schematic roadmap already being implemented by the EU Institutions has focused on efforts to help alleviate consumers of the “norovirus burden”. A short list of the ongoing efforts (certainly not exhaustive) concerning the “norovirus foodborne plight” on the part of the EU is the following: (i) more surveillance data to be collected from Member States (MS) from the European baseline survey that CEFAS, the respective EURL has already put in place, (ii) more intensified high-caliber research on “norovirus” from the EU HORIZON 2020 research project COMPARE [41] already underway, (iii) a risk-based microbiological criteria proposal to be put forward from EFSA, which will enable HACCP implementation options for norovirus in food of (non) animal origin, and (iv) for legislation to be enforced for all stakeholders to comply with and all Competent Authorities (both in EU MS and in TC) to abide by.

Other EU institutions with a long history such as the Food and Veterinary Office or FVO (via its constant interaction with the RASFF), contribute to ensuring that all RASFF member countries have attained the same EU legislation enforcement level at all times and thus have ultimately reached the harmonization objective regarding the defined EU food safety goals. Based on its mandate, and concerning the risk of “norovirus” in soft fruits and raspberries, the EU FVO has realized one visit to China (DG (SANCO) 2013-6682) and another one in Serbia (DG (SANCO) 2013-6660). Recommendations were made to the respective Competent Authorities and corrective action was undertaken on the part of the latter. This is, at large, the mode of action that the EU FVO is mandated to exercise its role, both towards EU Member States and Third Countries that export to the EU. The EU FVO has aided significantly in the areas of assessment of the control systems in place to manage the risk of contamination by “norovirus” in Third Countries. Another EU institution that contributes greatly to the “norovirus” epidemiological follow-up is the ECDC. Finally, the EU RASFF through its active participation in The International Food Safety Authorities Network or INFOSAN aims to strengthen global food safety response capabilities to ensure safer food for the entire world.

The above-mentioned multi-faceted approach seems like the coherent and well-designed way forward that should be followed, if we are to place “norovirus” in the context of legislated enforcement of microbiological (either process hygiene or food safety) criteria and through this path to secure that “norovirus” in food is officially monitored and regulated and risk management options are available. Overall, it becomes apparent that the EU as a whole, through its Institutions, streamlines all its efforts towards finally achieving risk-based microbiological criteria for “norovirus” in food, its ultimate objective being the protection of Public Health. Of course, more long-standing strategic aims would be the development of vaccines through international collaboration to avoid duplication of research efforts.

## 5. Conclusions

Food safety within the EU is a shared responsibility for all EU RASFF members acting as protectors of one another. The practical approach towards the shared protection capacity realization of all RASFF member countries is to respond to the specific threats in the specific foods that reach each one of them so that, at the end (provided that all members play their respective role efficiently), the entire European population is safe. This axiom is very well portrayed in the long-standing EU RASFF institution’s history of “norovirus”-related alert and border notifications. Through far-reaching, in-depth analyses of the EU RASFF database, emerging risks can be identified, changing trends in established foodborne infections can be closely monitored, and, ultimately, public health can be safeguarded against foodborne infections more efficiently (as is the case of “nororivus”), and could provide the information (of potential avail) to interested parties for future risk analysis efforts on “norovirus”. The international transmission potential of noroviruses is enhanced already by the ever growing human population, the international travel and the increasing globalization in food distribution [42]. This new era for “norovirus” will have to be resolved with the “one health” approach, where human and veterinary medicine collaborate to offer the desired high level of health protection to the world’s consumers.

## Figures and Tables

**Table 1:** Alert and border rejection notifications * for “norovirus” in food generally, and in the implicated food product categories from 1979 until 31 August 2017 in the European Union Rapid Alert System for Food and Feed (EU RASFF) database.

Types of Notifications	Food	Food + Fruits and Vegetables	Food + Bivalve Molluscs and Products Thereof	Food + Crustaceans and Products Thereof	Food + Molluscs and Products Thereof (Obsolete)
All types of notifications	219	63	143	7	5
Alert	107	46	52	5	4
-official control on the market		14	16	0	2
-company’s own check		6	5	0	0
-consumer complaint		3	2	0	1
-food poisoning		23	29	3	0
Border rejection	42	5	37	0	0

* Other types of notifications not shown here (e.g., information, information for attention, information for follow-up are not included in the scope of this work’s research).

**Table 2:** Countries/Organizations involved in alert and border rejection notifications * implicating “norovirus” in the two food product categories under examination from 1979 until 31 August 2017 in the EU RASFF database.

Types of Notifications	Organizations Involved		Countries Involved *		
	INFOSAN	Commission Services (CS)	0–3	4–9	10–19
Alert notifications	14	15	56	37	7
-bivalve molluscs	3	10	41	16	4
-fruits and vegetables	11	5	22	21	3
Border rejection notifications	0	8	42	0	0
-bivalve molluscs	0	8	37	0	0
-fruits and vegetables	0	0	5	0	0

* excluding INFOSAN and/or Commission Services (CS).

**Table 3:** Alert and border rejection notifications (according to risk decision) for all types of food product categories, concerning “norovirus” until 31 August 2017, in the EU RASFF.

**Alert Notifications Made on the Basis of Risk Decision:**	**“Bivalve Molluscs and Products Thereof”**	**“Fruits and Vegetables”**
serious	36	29
undecided	25	17
**Border Rejection Notifications Made on the Basis of Risk Decision:**		
serious	29	3
not serious	1	0
undecided	7	2

**Table 4:** Relative (%) frequency of citation of various “subject” notifications in alert notifications in “food” within the “pathogenic microorganisms” hazard category in the EU RASFF database (until 31 August 2017).

Relative (%) frequency of “subject” citation in alert notifications	Notifications on “Subject”		
	“Norovirus”	“*Listeria*”	“*Salmonella*”
Alert notifications+“subject”/All types of notifications+“subject”	107/219 (48.85%)	804/1360 (59.11%)	1653/4683 (35.29%)
Alert notifications+FP *+”subject”/Alert notifications +”subject”	55/107 (51.40%)	8/804 (0.99%)	52/1653 (3.14%)
Alert notifications+FP+”subject”/Alert notifications+FP	55/186 (30.30%)	8/186 (4.30%)	52/186 (26.89%)
Alert notifications+“serious” RD *+”subject”/Alert notifications+”subject”	65/107 (60.74%)	266/804 (33.08%)	582/1653 (35.20%)

* RD = risk decision, FP = food poisoning.

## References

[1] Regulation (EC) No 178/2002 of the European Parliament and of the Council of 28 January 2002 Laying Down the~General Principles and Requirements of Food Law, Establishing the~European Food Safety Authority and Laying Down Procedures in Matters of Food Safety Article 50. http://eur-lex.europa.eu/legal-content/EN/TXT/PDF/?uri=CELEX:32002R0178&from=EN.

[2] European Commission Commission Regulation (EU) No 16/2011 of 10 January 2011 Laying Down Implementing Measures for the Rapid Alert System for Food and Feed. http://eur-lex.europa.eu/legal-content/EN/TXT/PDF/?uri=CELEX:32011R0016&from=EN.

[3] De Wit M.A., Koopmans M.P., Kortbeek L.M., van Leeuwen N.J., Bartelds A.I., Van Duynhoven Y.T. (2001). Sensor, a population-based cohort study on gastroenteritis in the Netherlands: incidence and etiology. Am. J. Epidemiol..

[4] Kroneman A., Verhoef L., Harris J., Vennema H., Duizer E., van Duynhoven Y., Gray J., Iturriza M., Bottiger B., Falkenhorst G. (2008). Analysis of integrated virological and epidemiological reports of norovirus outbreaks collected within the foodborne viruses in Europe network form 1 July 2001 to 30 June 2006. J. Clin. Microbiol..

[5] Newell D.G., Koopmans M., Verhoef L., Duizer E., Aidara-Kane A., Sprong H., Opsteegh M., Langelaar M., Threfall J., Scheutz F. (2010). Food-borne diseases-The challenges of 20 years ago still persist while new ones continue to emerge. Int. J. Food Microbiol..

[6] Le Guyader F.S., Mittelholzer C., Haugarreau L., Hedlund K.O., Alsterlund R., Pommepuy M., Svensson L. (2004). Detection of noroviruses in raspberries associated with a gastroenteritis outbreak. Int. J. Food Microbiol..

[7] Wang J., Deng Z. (2012). Detection and forecasting of oyster norovirus outbreaks: Recent advances and future perspectives. Mar. Environ. Res..

[8] Bon F., Ambert-Balay K., Giraudon H., Kaplon J., Le Guyader S., Pommepuy M., Gallay A., Vaillant V., de Valk H., Chikhi-Brachet R. (2005). Molecular epidemiology of calicivirases detected in sporadic and outbreak cases of gastroenteritis in France from December 1998 to February 2004. J. Clin. Microbiol..

[9] Westrell T., Dusch V., Ethelberg S., Harris J., Hjertqvist M., Jourdan-da Silva N., Koller A., Lenglet A., Lisby M., Vold L. (2010). Norovirus outbreaks linked to oyster consumption in the United Kingdom, Norway, France, Sweden and Denmark. Eurosurveillance.

[10] De Graaf M., Villabruna N., Koopmans M.P.G. (2017). Capturing norovirus transmission. Curr. Opin. Virol..

[11] Cotton M., Koopmans M. (2016). Next-generation sequencing and norovirus. Future Virol..

[12] Polo D., Schaeffer J., Fournet N., Le Saux J.-C., Parnaudeau S., McLeod C., Le Guyader S. (2016). Digital PCR for quantifying Norovirus in oysters implicated in outbreaks, France, Emerg. Infect. Dis..

[13] Hewitt J., Croucher D., Kaas L., Lang J. Quantitative analysis of norovirus outbreak related shellfish samples using digital PCR and qPCR. Proceedings of the 11th International Conference on Molluscan Shellfish Safety (ICMSS) 2017.

[14] Fraisse A., Coudray-Meunier C., Martin-Latil S., Hennechart-Collette C., Delannoy S., Fach P., Perelle S. (2017). Digital RT-PCR method for hepatitis A virus and norovirus quantification in soft berries. Int. J. Food Microbiol..

[15] Le Guyader F.S., Bon B., DeMedici D., Parnaudeau S., Bertone A., Crudeli S., Doyle A., Zidane M., Suffredini E., Kohli E. (2006). Detection of multiple noroviruses associated with an international gastroenteritis outbreak linked to oyster consumption. J. Clin. Microbiol..

[16] Atmar R.L. Advances in Cultivation of Human Norovirus-Where we are now?. Proceedings of the 11th International Conference on Molluscan Shellfish Safety (ICMSS) 2017.

[17] Ettayebi K., Crawford S.E., Murakami K., Broughman J.R., Karandikar U., Tenge V.R., Neill F.H., Blutt S.E., Zeng X.L., Qu L. (2016). Replication of human noroviruses in stem cell-derived human enteroids. Science.

[18] NORONET. http://www.rivm.nl/en/Topics/N/NoroNet.

[19] RIVM. http://rivm.nl/en/Documents_and_publications/Common_and_Present/Newsmessages/2017/Norovirus_leading_cause_food_related_outbreaks.

[20] Siebenga J.J., Vennema H., Zheng D.P., Vinje J., Lee B.E., Pang X.L., Ho E.C., Lim W., Choudekar A., Broor S. (2009). Norovirus illness is a global problem: emergence and spread of norovirus GII.4 variants, 2001–2007. J. Infect. Dis..

[21] European Food Safety Authority (EFSA), Panel on Biological Hazards (BIOHAZ) (2011). Scientific Opinion on an update on the present knowledge on the occurrence and control of foodborne viruses. EFSA J..

[22] European Food Safety Authority (2012). Norovirus (NoV) in oysters: Methods, limits and control options. EFSA J..

[23] European Food Safety Authority, EFSA BIOHAZ Panel (EFSA Panel on Biological Hazards) (2014). Scientific Opinion on the risk posed by pathogens in food of non-animal origin. Part 2 (Salmonella and Norovirus in berries). EFSA J..

[24] European Food Safety Authority, EFSA BIOHAZ Panel (EFSA Panel on Biological Hazards) (2015). Scientific opinion on the evaluation of heat treatments, different from those currently established in the EU legislation, that could be applied to live bivalve molluscs from B and C production areas, that have not been submitted to purification or relaying, in order to eliminate pathogenic microorganisms. EFSA J..

[25] European Food Safety Authority (2016). Scientific report on technical specifications for a European baseline survey on norovirus in oysters. EFSA J..

[26] European Food Safety Authority (EFSA), European Centre for Disease Prevention and Control (ECDC) (2016). The European Union summary report on trends and sources of zoonoses, zoonotic agents and food-borne outbreaks in 2015. EFSA J..

[27] Opinion of the Food Safety Authority of Ireland Scientific Committee. Risk Management of Norovirus in Oysters (December 2013). https://www.fsai.ie/publications_norovirus_opinion/.

[28] Abrahantes J.C., Richardson J., O’Mahony M., Pare A., Bruckers L., Johne R., Keaveney S., Ianni I., Lowther J., Suffredini E. European baseline survey of norovirus in oysters. Proceedings of the 11th International Conference on Molluscan Shellfish Safety (ICMSS) 2017.

[29] European Union Reference Laboratory for Monitoring Bacteriological and Viral Contamination of Bivalve Molluscs, CEFAS. https://eurlcefas.org/.

[30] Food and Veterinary Office, FVO. https://ec.europa.eu/food/audits_analysis_en.

[31] European Centre for Disease Control and Prevention ECDC. https://ecdc.europa.eu/en/norovirus-infection.

[32] The International Food Safety Authorities Network (INFOSAN), Progress Report 2004–2010. http://www.fao.org/3/a-i2002e.pdf.

[33] Food Standards Agency (FSA) Proceedings of the Food Standards Agency’s Foodborne Viruses Research Conference. https://www.food.gov.uk/sites/default/files/multimedia/pdfs/publication/foodborne-virus-2013.pdf.

[34] Leuschner R.G.K., Hristova A., Robinson T., Hugas M. (2013). The Rapid Alert System for Food and Feed (RASFF) database in support of risk analysis of biogenic amines in food. J. Food Compos. Anal..

[35] European Union Rapid Alert System for Food and Feed, RASFF. https://ec.europa.eu/food/safety/rasff_en.

[36] Attachment 6. Risk Profile of Norovirus in Bivalve Molluscan Shellfish (Netherlands), CX/FH 06/38/10. 54–62. http://www.nihs.go.jp/hse/food-info/microbial/noro/Noro_riskprofile.pdf.

[37] Alfano-Sobsey E., Sweat D., Hall A., Breedlove F., Rodriguez R., Greene S., Pierce A., Sobsey M., Davies M., Ledford S.L. (2011). Norovirus outbreak associated with undercooked oysters and secondary household transmission. Epidemiol. Infect..

[38] Le Guyader F.S., Atmar R.L., Le Pendu J. (2012). Transmission of viruses through shellfish: When specific ligands come into play. Curr. Opin. Virol..

[39] Koopmans M., Duizer E. (2004). Foodborne viruses: an emerging problem. Int. J. Food Microbiol..

[40] Codex Alimentarius Commission CAC/GL 79-2012 (2012). Guidelines on the Application of General Principles of Food Hygiene to the Control of Viruses in Food.

[41] COMPARE EU HORIZON 2020 Project. http://www.compare-europe.eu/.

[42] De Graaf M., van Beek J., Koopmans M.P. (2016). Human norovirus transmission and evolution in a changing world. Nat. Rev. Microbiol..

